# Overexpression of HTRA1 Leads to Down-Regulation of Fibronectin and Functional Changes in RF/6A Cells and HUVECs

**DOI:** 10.1371/journal.pone.0046115

**Published:** 2012-10-08

**Authors:** Jingjing Jiang, Lvzhen Huang, Wenzhen Yu, Xi Wu, Peng Zhou, Xiaoxin Li

**Affiliations:** 1 Department of Ophthalmology, Peking University People's Hospital, Beijing, China; 2 Key Laboratory of Vision Loss and Restoration, Ministry of Education, Beijing, China; 3 Department of Ophthalmology, China-Japan Friendship Hospital, Beijing, China; 4 Department of Ophthalmology, Eye and ENT Hospital of Fudan University, Shanghai, China; University of Sassari, Italy

## Abstract

Multiple genetic studies have suggested that high-temperature requirement serine protease (HTRA1) is associated with polypoidal choroidal vasculopathy (PCV). To date, no functional studies have investigated the biological effect of HTRA1 on vascular endothelial cells, essential vascular components involved in polypoidal vascular abnormalities and arteriosclerosis-like changes. In vitro studies were performed to investigate the effect of HTRA1 on the regulation of fibronectin, laminin, vascular endothelial growth factor (VEGF), platelet derived growth factor receptor (PDGFR) and matrix metalloparoteinases 2 (MMP-2) and the role of HTRA1 in choroid-retina endothelial (RF/6A) and human umbilical vein endothelial (HUVEC) cells. Lentivirus-mediated overexpression of HTRA1 was used to explore effects of the protease on RF/6A and HUVEC cells in vitro. HTRA1 overexpression inhibited the proliferation, cell cycle, migration and tube formation of RF/6A and HUVEC cells, effects that might contribute to the early stage of PCV pathological lesions. Fibronectin mRNA and protein levels were significantly down-regulated following the upregulation of HTRA1, whereas the expressions of laminin, VEGF and MMP-2 were unaffected by alterations in HTRA1 expression. The decreased biological function of vascular endothelial cells and the degradation of extracellular matrix proteins, such as fibronectin, may be involved in a contributory role for HTRA1 in PCV pathogenesis.

## Introduction

Polypoidal choroidal vasculopathy (PCV) is a major cause of serosanguinous maculopathy, a condition associated with a reduction of vision in the elderly Asian population. PCV was first described by Yannuzzi and associates in 1990 as an idiopathic PCV presenting polypoidal and choroidal vascular lesions [1]. The incidences of PCV among Chinese and Japanese populations with fundus characteristics of neovascular age-related macular degeneration (nAMD) are 24.5% and 54.7%, respectively, which are much higher than the incidence noted in Caucasians [2], [3], [4]. Distinct from the clinical features of AMD, PCV is characterized on indocyanine green (ICG) by a network of branching abnormal choroid vessels and polypoidal vascular dilations. The etiology and pathogenesis of PCV are largely unclear.

HTRA1 (high-temperature requirement factor A-1), a member of the high-temperature requirement A family of serine proteases, is ubiquitously expressed in various normal adult human tissues, such as the epidermis, vascular endothelia and neuronal cells [5]. HTRA1 mutations have been associated with familial ischemic cerebral small-vessel disease (CARASIL), which is characterized by non-hypertensive cerebral small-vessel arteriopathy [6]. Previous studies have reported raised levels of HTRA1 expression in drusen, abnormal retinal pigment epithelial (RPE) cells and choroidal neovascular membranes [7], [8], [9].

It has been well established that variation in HTRA1 has a strong genetic effect on AMD, a disease sharing certain common environmental risk factors and genetic determinants with PCV [10]. The functional single nucleotide polymorphism (SNP) rs11200638, located in the promoter region of the HTRA1 gene within the 10q26 locus, has been identified as one of the most closely associated AMD risk factors [7], [11]. Recently, an increasing number of studies have investigated the possible association of PCV in Asian populations with rs11200638 in HTRA1 [12], [13], [14], [15]. However, a summary of the genetic effects of this variant on the susceptibility to PCV has not been reviewed.

The mechanism by which HTRA1 instigates the ocular tissue abnormalities of AMD has been discussed in functional studies. Many of these studies suggest a link between HTRA1, fibronectin and stabilization of the extracellular matrix in AMD pathogenesis [8], [16], [17]. As an important signal protein promoting angiogenesis, VEGF should also be studied to determine if there is any connection with HTRA1 in AMD [18], [19]. Thus, we speculated that the expression of HTRA1 could be associated with fibronectin and VEGF and that it could ultimately be involved in the regulation of PCV. Further studies evaluating the influence of HTRA1 on vascular endothelial cell and protein-protein interactions are needed.

## Materials and Methods

### Construction and identification of the HTRA1 expression plasmid

The primers targeting the human HTRA1 gene were synthesized based on a cDNA library (Genechem,Shanghai, China), and the sequences were as follows: a) HTRA1-AgeI-F 5′-GAGGATCCCCGGGTACCGGTCGCCACCATGCAGATCCCGCGCGCCGCTCTTCTC-3′, and HTRA1-AgeI-R 5′-AGTCCATGGTGGCGACCGGTGGGTCAATTTCTTCGGGAATC-3′. These primers contain the complement bases, the AgeI restriction site and a partial sequence of the 3′ end of the target gene, which is used for isolating the target gene by polymerase chain reaction (PCR). b) HTRA1-SEQF 5′-CTCTCCGGCCCTCGCCCTGTC-3′. This primer was located in the coding sequence of the HTRA1 gene and used in colony PCR to identify positive transformants. c) EGFP-N-R 5′-CGTCGCCGTCCAGCTCGACCAG-3′. This primer was used in PCR to identify transformants and in sequencing. Each of these fragments was amplified, digested with AgeI and then ligated into the pGC-FU-3FLAG vector for 15 min at 23°C and 42°C. The recombinant was transformed into competent *Escherichia coli* cells treated with calcium chloride and incubated for 16 h at 37°C. PCR conditions were as follows: 94°C for 5 min, 30 cycles of 94°C for 30 s, 55°C for 30 s, 72°C for 2 min and finally 72°C for 10 min. Positive clones, as confirmed by PCR, were chosen for sequencing. The plasmids with the correct sequence were transfected into 293T cells, and the expression of the confluency protein was observed under a fluorescence microscope. Western blotting was applied to detect the expression of the confluency protein. The virus titer was determined by quantitative real-time PCR.

### Cell culture and lentivirus transduction

A rhesus monkey choroid-retina endothelial cell line (RF/6A) and human umbilical vein endothelial cells (HUVECs) were obtained from the American Tissue Culture Collection(Manassas, VA) and the cell bank of the Chinese Academy of Science (Shanghai, China), respectively. The cells were cultured in Dulbecco's Modified Eagle Medium/F12 (Gibco, Grand Island, NY) with 10% fetal bovine serum (FBS) at 37°C in 5% CO_2_ and 95% humidity. When the RF/6A cells and HUVEC cells were approximately 50% confluent in fresh serum-free medium, they were transiently transduced with control lentivirus or HTRA1-overexpression lentivirus at MOIs (multiplicities of infection) of 100 and 10, respectively. The cells were further cultured in DMEM with 10% FBS after infecting for 4 hours and then selected using 200 µm/ml puromycin. The stable overexpression lines were established when more than 90% of the transduced cells were found to strongly express GFP under fluorescent microscopy. The RF/6A cells and HUVEC cells were both divided into 3 groups: control(C), the lentiviral vector control group (NC), and the HTRA1-transfected group (HTRA1).

### RNA isolation and real-time RT-PCR

The total RNA was isolated from RF/6A cells and HUVEC cells using TRIzol reagent (Invitrogen, Carlsbad, CA) according to the manufacturer's instructions. RNA extract (2 µl) was reverse-transcribed into cDNA in a total reaction volume of 20 µl using a RevertAid™ First Strand cDNA Synthesis Kit (Fermentas, Burlington, Canada). Real-time quantitative PCR was performed using IQ Supermix (Bio-Rad, Hercules, CA), with 20-µl reaction mixtures containing 2 µl cDNA, 7.2 µl sterilized water, 10 µl SYBR Green Real-time PCR Master Mix (TaKaRa, Japan), and 0.8 µl of primer (10 µM). The HTRA1, VEGF and fibronectin amplification signals were normalized to β-actin expression and evaluated using the equation: fold change = 2^−ΔΔct^. The primer sequences used in this study were as follows: HTRA1, forward primer 5′-GGGAAAGCACCCTGAACAT-3′, reverse primer 5′-CCAGAGTCCTCATCCGTCAT-3′; VEGF, forward primer 5′-AGTTCCACCACCAAACATGC-3′, reverse primer 5′-TGAAGGGACACAACGACACA-3′; fibronectin, forward primer 5′-GGGAGCCTCGAAGAGC-3′, reverse primer 5′-AACAAGTACAAACCAACGCA-3′; and β-actin, forward primer 5′-CTTAGTTGCGTTACACCCTT-3′, reverse primer 5′-CCTTCACCGTTCCAGTTT-3′. Laminin, forward primer 5′-TAACTGGTGGCAAAGTCC-3′, reverse primer 5′-AGAGAACGCTCCAAAATC-3′; MMP-2, forward primer 5′-AACTTCTTCCCTCGCAAG-3′, reverse primer 5′-TGTCTGCCTCTCCATCAT-3′; PDGFR, forward primer 5′-TCAGACAGAAGAGAATGAGC-3′, reverse primer 5′-GTCTCGGGATCAGTTGTG-3′.

### Protein extraction and western blot analysis

RF/6A cells and HUVEC cells were washed three times with ice-cold phosphate-buffered saline (PBS, 4°C, pH 7.4) for 5 min at room temperature and prepared using a protein extraction kit and a protease inhibitor kit (Pierce, Rockford, IL). The supernatant was collected and the protein content of each lysate was determined using a BCA Protein Assay Kit (Tianlai Shengwu Jishu, Tianlai, China) according to the manufacturer's instructions. Equal amounts (20 µg) of protein were electrophoresed on a 10% sodium dodecyl sulfate (SDS) polyacrylamide gel and transferred onto a 0.22 µm PVDF membrane (Millipore). The primary antibodies used to probe the membranes included anti-HTRA1 (1∶300; Abcam, Cambridge, UK, Cat No. ab38610), anti-Fibronectin (1∶1800; Abcam, Cambridge, UK, Cat No. ab2413), anti-laminin (1∶600; Abcam, Cambridge, UK, Cat No. ab11575) and anti-β-actin (1∶3000; Boster, Wuhan, China). The membranes were washed and incubated with peroxidase-conjugated secondary antibodies (1∶5000; Boster, China). Enhanced chemiluminescence Western blotting detection reagents (Pierce) were used to detect HTRA1, fibronectin and laminin protein levels.

### Measurement using an enzyme-linked immunosorbent assay

The RF/6A and HUVEC cells (untransfected and stably transfected cells) were seeded in 24-well plates. Next, the culture supernatants were separately collected at 12, 24, 48 ,72 h and 96 h and were centrifuged to eliminate cellular fragments. The concentration of the secreted VEGF and MMP-2 in the media was determined with commercially available enzyme-linked immunosorbent assay (ELISA) kits (Boster, Wuhan, China) according to the manufacturer's protocol. All samples were assayed in triplicate and the mean values were calculated and expressed as pg VEGF or MMP-2/10^5^ cells.

### In vitro cell proliferation assay

To determine the role of pGC-FU-3FLAG-HTRA1 transfection in the proliferation of RF/6A cells and HUVECs, a 3-[4,5-dimethylthiazol-2-yl]-2,5-diphenyltetrazoliumbromide (MTT; Roche, Molecular Biochemicals, Mannheim, Germany) assay was used to measure cell proliferation over 4 consecutive days. RF/6a cells and HUVECs, both divided into three groups, were plated at a density of 1×10^4^ cells per well in 96-well culture plates. After a 24-h incubation, MTT was added, and the cells were incubated for an additional 4 h. Formazan crystals were then dissolved by the addition of dimethyl sulfoxide (DMSO, 100 µl/well). The absorbance at 570 nm was measured using an ELISA plate reader (Dynatech Medica, Guernsey, UK). Each group was measured using a modified MTT assay at 24, 48, 72 and 96 h, and the experiment was performed at least three times.

### Flow cytometry

Each group of cells was seeded in 6-well plates for 48 h. The cells were detached using ethylene diamine tetraacetic acid (EDTA), washed in ice-cold PBS (4°C), and treated with the BD Cycletest™ Plus DNA Reagent Kit (Becton Dickinson) according to the manufacturer's protocol. Samples were analyzed using a FACSCalibur flow cytometer (Becton Dickinson, Franklin Lakes, NJ). Three samples were used in one experiment and each experiment was repeated.

### Cell migration

**A** migration assay was performed as described previously [21]. Briefly, 2×10^4^ cells were placed in the upper chamber in a final volume of 200 µl of serum-free medium, and 10% FBS then was added to the bottom chamber for a final volume of 600 µl. All migration assays were conducted for 4 h at 37°C. At the end of the assay, the cells were fixed in 95% ethanol and stained with hematein for 25 min. The remaining cells were wiped away with a cotton swab, and the membrane was imaged. The numbers of cells from five random fields of view were counted.

### Tube formation

A tube formation assay was performed to investigate the effect of pGC-FU-3FLAG-HTRA1 transfection on RF/6A cells and HUVECs as described previously [22]. Aliquots (150 µl) of matrigel solution were poured into the 48-well plates (repeated twice), and the plates were incubated at 37°C for 1 h in a 5% CO_2_ incubator to form a matrigel. Three groups of RF/6A cells and HUVECs were seeded on the matrigel and cultured in DMEM medium for 14 h. Five random fields of the networks in the matrigel were counted and photographed under a microscope.

## Results

### Identification of recombinant plasmid pGC-FU-3FLAG-HTRA1

The positive clones were identified after PCR amplification; the size of the PCR product was 1486 bp. The detected sequence was identical to the known HTRA1 sequence in GenBank (Gene ID 5654). A western blot revealed a 55-kDa band in cell extracts, which was in accordance with the expected size of an HTRA1–Flag confluency protein (53 kDa+2 kDa = 55 kDa). These results indicate that the HTRA1 recombinant plasmid was successfully expressed in RF/6A and HUVEC cells and suggested that these cells were successfully transduced with lentivirus.

### Overexpression of HTRA1 by stable transfection of pGC-FU-3FLAG-HTRA1

**Figure 1:**
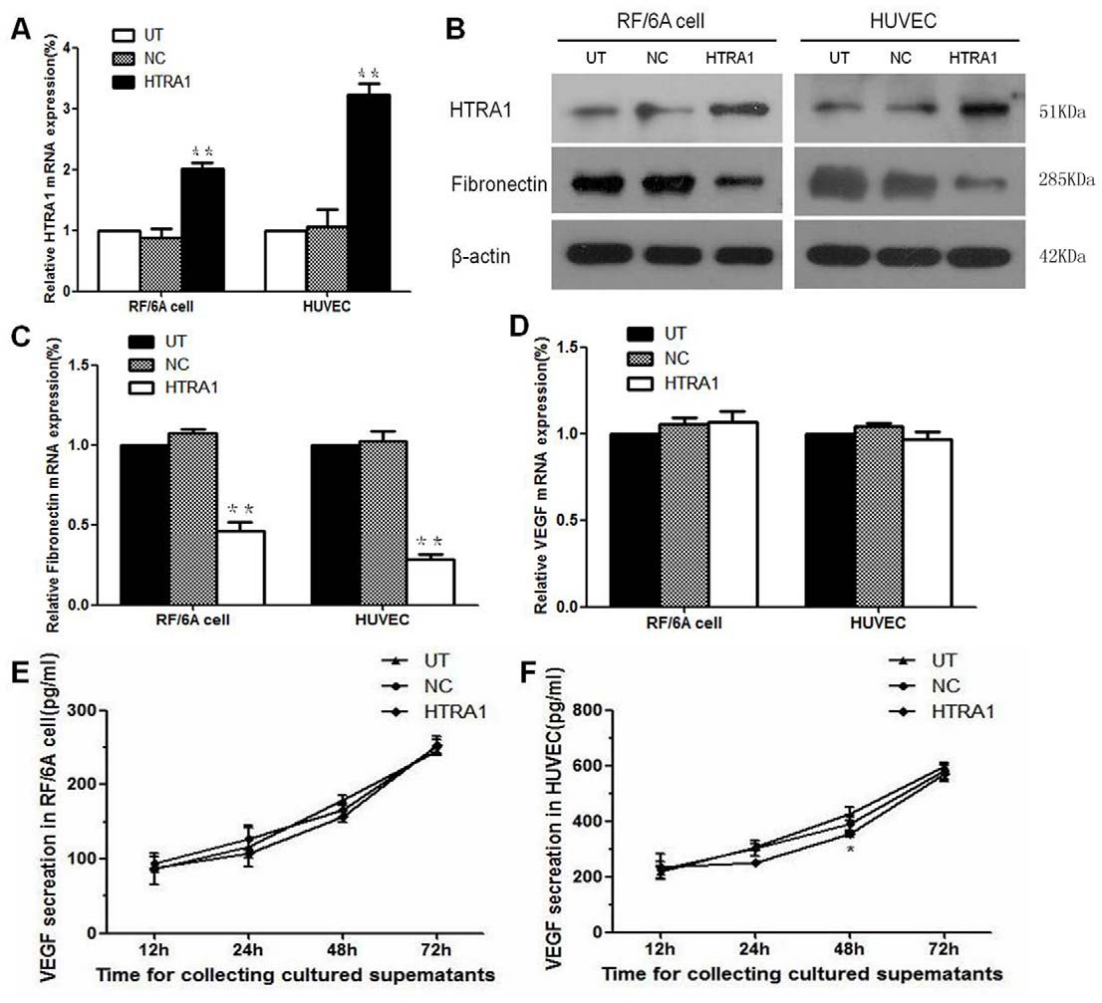
Effect of pGC-FU-3FLAG-HTRA1 on HTRA1, fibronectin and VEGF expression in RF/6A cells and HUVECs as determined by real-time RT-PCR and western blotting or ELISA. Messenger RNA (A) and protein (B) expression of HTRA1 in RF/6A cells and HUVECs transfected with pGC-FU-3FLAG-HTRA1 were increased when compared with the control groups (**P<0.01). The fibronectin mRNA (C) and protein (B) levels were reduced in HTRA1-overexpressing cells (**P<0.01), while no significant differences were noted in the expression levels of VEGF mRNA (D) and secreted protein, except for a slight decrease in protein levels in cultured HUVEC supernatants at 48 hours (*P<0.05).

Total mRNA and protein extracts were prepared from untransfected control (UT), lentiviral vector control (NC) and HTRA1-transfected (HTRA1) RF/6A cells and HUVECs. The HTRA1 mRNA and protein expression levels in these cells were determined by real-time RT-PCR and Western blot assays, respectively. Real-time RT-PCR demonstrated that the mRNA levels of HTRA1 protein expression in the transfectants containing pGC-FU-3FLAG-HTRA1 were increased 2-fold and 3-fold, respectively, compared with untreated control RF/6A cells and HUVECs, which was consistent with the increase of HTRA1 protein expression (p<0.01; Figure 1A, Figure 1B). There was no significant difference between the cells transfected with the control lentiviral vector and the untransfected cells (p>0.05). These results indicate the stable transfection of pGC-FU-3FLAG-HTRA1 up-regulated HTRA1 expression in both RF/6A cells and HUVECs.

### Effect of HTRA1 on fibronectin, laminin , VEGF and MMP-2 protein expression

**Figure 2:**
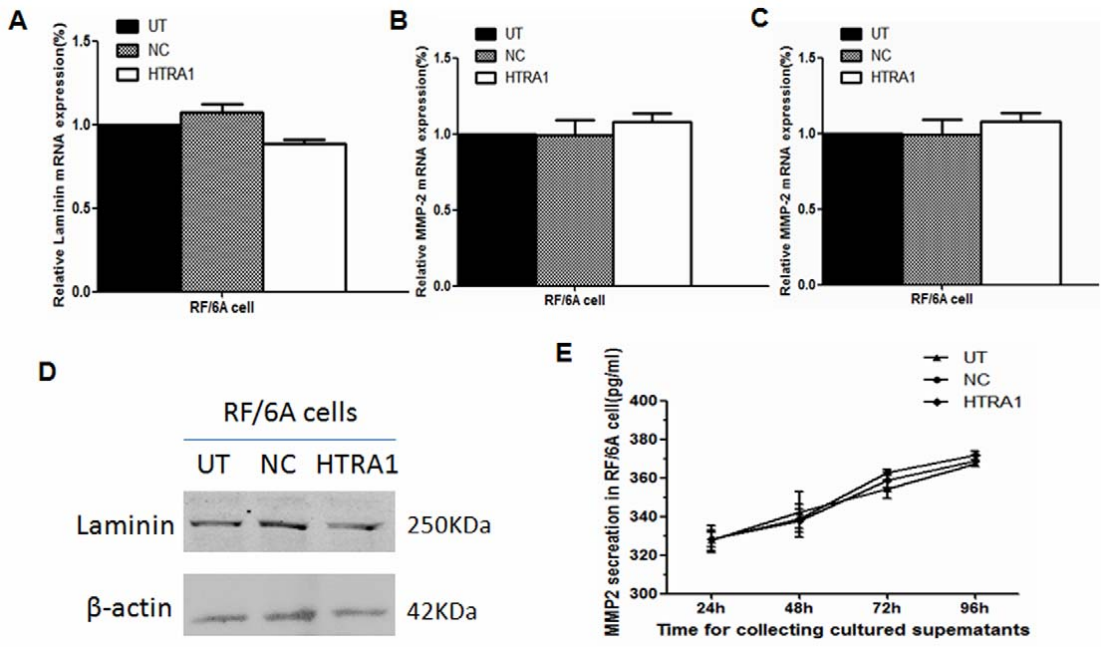
Effect of pGC-FU-3FLAG-HTRA1 on laminin, PDGFR and MMP-2 expression in RF/6A cells as determined by real-time RT-PCR and western blotting or ELISA. Messenger RNA expression of laminin, PDGFR and MMP-2 in RF/6A cells transfected with pGC-FU-3FLAG-HTRA1 showed no significant changes compared with the control groups (A, B, C). No significant differences were noted in the protein expression levels of laminin and MMP-2.

Fibronectin plays an important role in maintaining dynamic equilibrium in the extracellular matrix and is associated with vascular development [23], [24]. We investigated whether the overexpression of HTRA1 induced by pGC-FU-3FLAG-HTRA1 could affect fibronectin and angiogenesis-associated genes, such as VEGF. The levels of fibronectin mRNA and protein in the UT, NC, and HTRA1-transfected cells were determined by real-time RT-PCR and western blot. When compared with the control groups, HTRA1 transfection led to a significant 54% decrease (p<0.01) and a 72% (p<0.01) decrease in fibronectin mRNA levels in RF/6A cells and HUVECs, respectively. The results were consistent with the decrease of fibronectin protein expression (p<0.01; Figure 1C,Figure 1B). We also analyzed the levels of VEGF mRNA in the cells and secreted VEGF protein in the conditioning media by real-time RT-PCR and ELISA assays. There were no significant differences between HTRA1-transfected groups and control groups of RF/6A cells and HUVECs in either mRNA (Figure 1D) or protein levels of VEGF secretion in the cells or in the culture medium (P>0.05; Figure 1E, Figure 1F), with the exception of a slight decrease of protein level in cultured HUVEC supernatants collected after by 48 h of incubation (P<0.05). In addition, the mRNA level of laminin, PDGFR and MMP-2 had no significant differences between groups in RF/6A cells (Figure 2A, Figure 2B and Figure 2C). The protein levels of laminin and MMP-2 showed no significant changes in HTRA1-transfected groups and control groups of RF/6A cells (Figure 2D, Figure 2E).

### HTRA1 regulates RF/6A cells and HUVECs proliferation, cell cycle and cell migration

**Figure 3:**
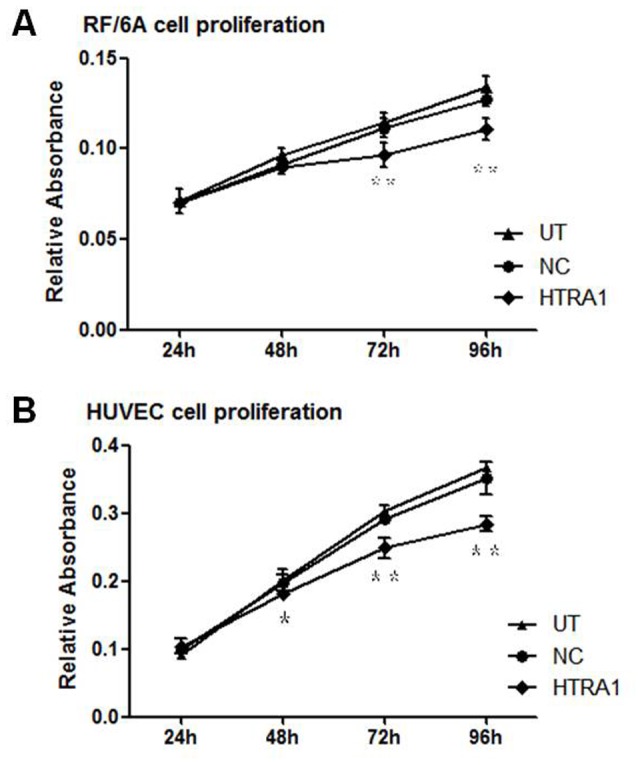
Effect of HTRA1 on the proliferation of RF/6A cells and HUVECs. Proliferation of RF/6A cells (A) and HUVECs (B) was measured with an MTT assay at 24, 48, 72 and 96 h. The values are listed as the means±SD of at least three independent experiments. Compared with the control groups, RF/6A cell proliferation was reduced by 7% at 48 h and this reduction peaked at 17% on the fourth day (**p<0.01); HUVEC proliferation was reduced by 10% (*P<0.05) at 48 h, and this reduction peaked at 23% (**P<0.01).

To determine the effect of HTRA1-induced RF/6A and HUVEC proliferation, the metabolic activities of UT, NC and HTRA1 cells were quantified using the MTT assay. RF/6A cell proliferation was reduced by 7% at 48 h compared with NC cells (P>0.05) and the reduction peaked at 17% on the fourth day (p<0.01). HUVEC proliferation was reduced by 10% at 48 h compared with NC cells (P<0.05) and the reduction peaked at 23% on the fourth day (p<0.01) (Figure 3).

**Figure 4:**
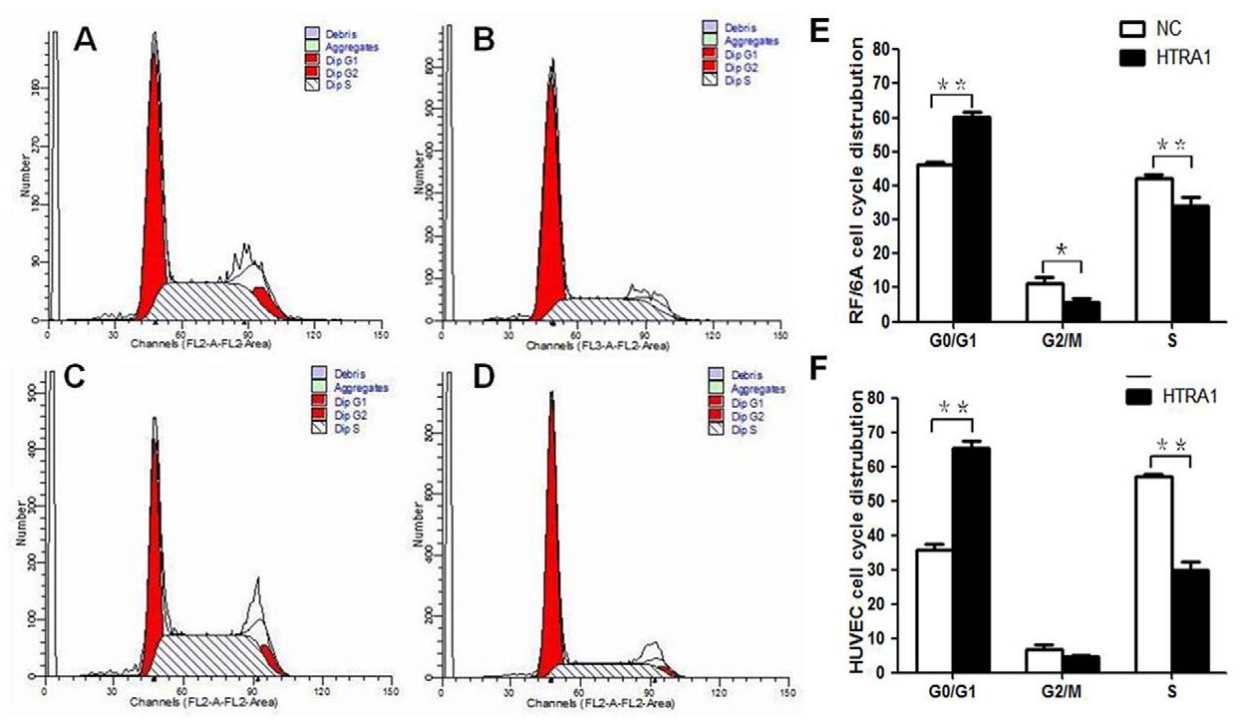
Effect of HTRA1 on the cell cycles of RF/6A cells and HUVECs. Flow cytometric analysis demonstrated that the fraction of G1-phase cells increased and the proportion of S-phase cells decreased in the RF/6A cells (A and B) and HUVECs (C and D) after increased expression of HTRA1. The proportions of G0/G1, G2, and S phase cells decreased in HTRA1-transfected RF/6A cells compared to control RF/6A cells transduced with the lentiviral vector (E, *p<0.05, **p<0.01). The proportions of G0/G1-and S-phase cells were decreased in HTRA1-transfected HUVECs compared with the control HUVECs transduced with the lentiviral vector (F, **p<0.01).

Next, flow cytometric analysis was applied to analyze the effect of HTRA1 on RF/6A cells and HUVEC cycles. As shown in Figure 4, the percentage of RF/6A cells in G0/G1 phase increased from 46.27% (NC) to 58.83% (HTRA1), and the S-phase cells decreased from 42.29% (NC) to 34.17% (HTRA1). The percentage of HUVECs in G0/G1 phase increased from 35.97% (NC) to 65.32% (HTRA1) and the S-phase cells decreased from 57.10% (NC) to 28.61% (HTRA1). The difference between HTRA1-transfected RF/6A cells and NC RF/6A cells in G2/M phase was slight (11.44% versus 5.66%, p<0.05), whereas the difference between HTRA1-transfected HUVECs and NC HUVECs in G2/M phase was not significant (6.93% versus 4.74%, p>0.05).

**Figure 5:**
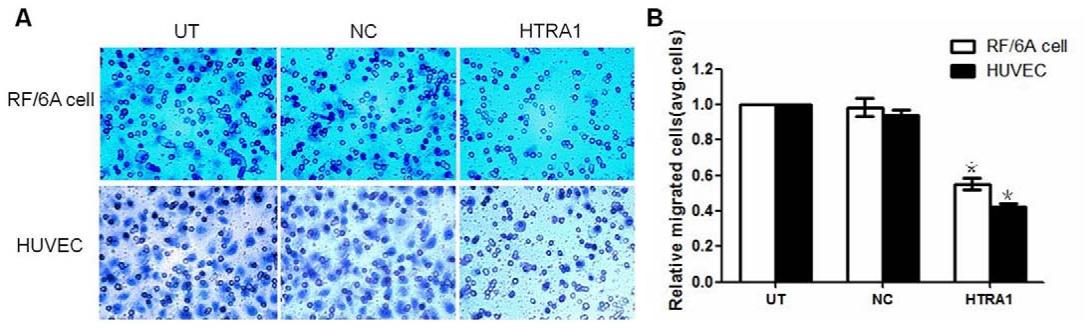
Effect of HTRA1 on the migration of RF/6A cells and HUVECs. The migratory activities of both cell lines were estimated based on the numbers of cells that had migrated through the filter of the chamber. The numbers of migrating cells in the HTRA1-transfected group were less than the number observed in the untransfected control group and the lentiviral vector control group (G, *p<0.01).

To explore the role of HTRA1 in the migration of RF/6A cells and HUVECs, we used a modified Boyden chamber in which these cells migrated through a porous membrane. The mean counts of migrating cells among the HTRA1 group of the RF/6A cells and HUVECs were significantly lower than in the NC group (p<0.05, Figure 5). In contrast, the mean counts of migrated cells in the NC and UT groups were not significantly different (p>0.05).

### HTRA1 has a slight effect on tube formation in RF/6A cells and HUVECs

**Figure 6:**
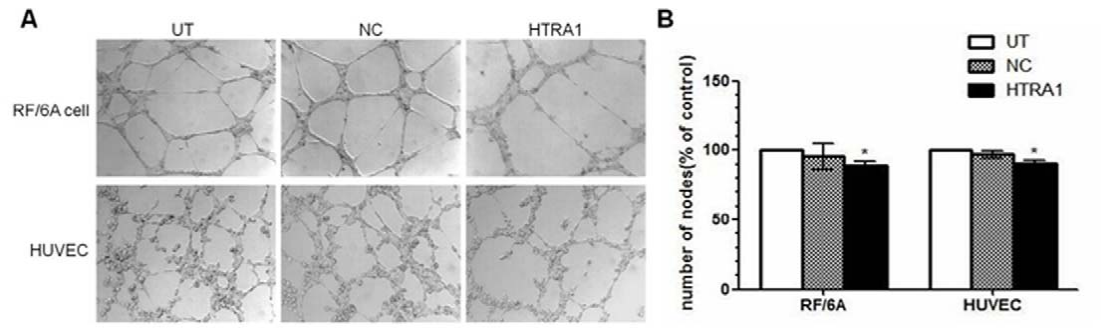
Effect of HTRA1 on tube formation in RF/6A cells and HUVECs. After 24 h of incubation, untransfected control cells (A) and lentiviral vector control group cells (B) formed well-organized, capillary-like structures, while the capacity of HTRA1-transfected group cells to organize was severely compromised (C, p<0.05).

To investigate whether HTRA1 could affect the angiogenic properties of RF/6A and HUVEC cells, we performed a capillary-like tube formation assay in matrigel. The HTRA1-transfected RF/6A cells and HUVECs both demonstrated little impairment of their capacities to form regular networks, and the differences between HTRA1-transfected cells and NC cells were slight in RF/6A cells (p<0.05, Figure 6) and HUVECs (p<0.05). There was no significant difference between the NC and UT groups.

## Discussion

A meta-analysis focused on the relationship between HTRA1 variants and PCV was performed as described previously [20] (Table S1). The meta-analysis indicated a 2.6-fold increase in susceptibility to PCV among persons with the A allele of the HTRA1 rs11200638 SNP. Compared to GG homozygotes, the AA homozygous and AG heterozygous variants carry a 6.4-fold and 1.8-fold increased risk for PCV, respectively (Table S2 and Figure S1). These data support the notion that the HTRA1 variant may be a risk factor for PCV. The functional SNP, located in the promoter region of the HTRA1 gene, has been reported to increase expression levels of HTRA1 [25], [26]. Using HTRA1 overexpression strategies, our experimental results show that this risk may result partly from decreased vascular endothelial cell biological function and degraded extracellular matrix proteins, such as fibronectin.

It has been well established that PCV lesions arise from the inner choroid vascular network and are characterized by polypoidal vascular abnormality, which implied that vascular endothelial cells may play an important role in the pathogenesis of PCV. HTRA1 has been reported in vascular endothelia, and some expression is found in the peripheral retinas [5], [27]. Moreover, proteins of the transforming growth factor β (TGF-β) family, which have been associated with the most-documented function of HTRA1, have multifaceted roles in vascular endothelial cells, depending on the type of cell and extracellular matrix [6], [28]. Dysregulation of TGF-β-family signaling results in hereditary vascular disorders [29]. However, previous studies investigating the functional effects of HTRA1 on PCV or AMD mainly focused on RPE cells or Bruch's membrane [8], [17], [18], [30], and potential effects on vascular endothelial cells have been only rarely observed.

In this study, we demonstrated that overexpression of HTRA1 induces varying degrees of reduction in the proliferation, cell cycle, migration and tube formation of RF/6A cells and HUVECs. The inhibition of proliferation was apparent in the HTRA1-transfected cells after 48 h. The cell growth of both RF/6A cells and HUVECs was also slowed due to a decreased progression past the G1/S checkpoint of the cell cycle. These impacts on vascular endothelial cells may be partly explained by the contributions of HtrA serine proteases to programmed cell death [31], [32]. Enhanced expression of HTRA1 markedly attenuates cell migration, whereas downregulation of HTRA1 promotes cell motility [33]. In addition, a crucial functional indicator of vascular endothelial cells, the tube formation capability of RF/6A cells and HUVECs, was degraded, as observed on matrigel. Thus, HTRA1 might play a role in maintaining the normal physiologic functions and homeostatic functions of RF/6A cells and HUVECs. Furthermore, normal vascular endothelial cells are thought to have atheroprotective functions [34]. Repeated or chronic endothelial cell loss and dysfunction might be the first event leading to atherosclerosis [35]. A key finding in the histopathological features of PCV is vessel hyalinization, which suggests an association with arteriosclerotic changes [10] In addition, HTRA1 has been suggested to play a critical role in the regulation of angiogenesis via TGF-β signaling [36]. Therefore, an excess of HTRA1 could have a detrimental effect on physiologic functions and homeostasis of vascular endothelial cells, which might lead to the destruction or dysfunction of vascular endothelium and might contribute to the early stage of PCV pathological lesions.

Fibronectin, as a major component of the vascular basement membrane and a mediator of extracellular matrix assembly, was found to be strongly associated with HTRA1 in ocular fundus lesions. Bajenaru et al. revealed that the loss of astrocytic fibronectin results in a mild vascular defect characterized by delayed vessel extension [37]. More abundant expression of fibronectin in the RPE of AMD donors was reported by An et al. [38]. Vierkotten et al. demonstrated that HTRA1 degrades fibronectin in RPE cells, and a higher level of expression of active fibronectin fragments was detected in HtrA1 transgenic mice [17]. In this study, we observed that fibronectin was expressed in both RF/6A cells and HUVECs and was significantly decreased by overexpressing HTRA1. The down-regulated fibronectin may impact the PCV lesion process by involving aneurysm or dilation of the abnormal vessels. Fibronectin has been reported to regulate the complement pathway [8]. Meanwhile, fibronectin fragments, produced by increased fibronectin digestion related to HTRA1, could act as a downstream protagonist of intraocular inflammation and catabolism [16]. The fibronectin fragments also have the capacity to degrade the stabilization of the extracellular matrix and to stimulate the release of cytokines and matrix metalloproteinases (MMPs) from RPE cells, which might further play a contributory role in PCV pathogenesis. HtrA1 or HtrA1-generated fibronectin fragments was reported be able to induce the expression of matrix metalloprotease 1 and matrix metalloprotease 3 [39]. Activities of MMP-2 is closely associated with the migratory capacity of cells. We did not find significant change of MMP-2 expression in different groups. Maybe the effects of HTRA1 on different types of matrix metalloprotease are different. Davide V et al showed that matrix metalloproteinase 2 regulate human aortic smooth muscle cell migration differently in young cells and aged cells [40]. Therefore we conclude that the over-expression of HTRA1 did not regulate the activation of MMP-2, and the migratory capacity changes in our study were not induced by MMP-2. Moreover, laminin is an essential basement membrane protein as fibronectin and plays a role in the stability or maturation of vessels and their barrier functions [41]. However, our results showed that HTRA1 has no effect on Laminin. The different results maybe reasoned form the property differences of fibronectin and Laminin [42].

The role of VEGF in the pathogenesis of PCV has been controversial in previous studies. Matsuoka et al. found that VEGF was strongly expressed in the tissues from an eye with PCV and in the vascular endothelial cells in human choroidal neovascular membranes [19]. In contrast, according to Nakashizuka et al., within PCV lesion histologic specimens, vascular endothelial cells were negative for VEGF [9]. In addition,, the interrelationship between VEGF and HTRA1 is also open to question. Ng et al. revealed that HTRA1 and VEGF were upregulated in fetal RPE cells during stress induction, but they do not mutually regulate their expression [18]. VEGF was up-regulated in transgenic mice overexpressing human HTRA1, as reported by Jones et al [32]. To explore if upregulation of HTRA1 has any effect on the expression levels of VEGF in RF/6A cells and HUVECs, we measured mRNA and secreted protein levels of HTRA1. There were no significant differences in VEGF expression levels between HTRA1-transfected groups and control groups of RF/6A cells and HUVECs, except for a slight down-regulation of protein level in cultured HUVEC supernatants collected at 48 h post-transfection. The difference between the two cell types may be attributable to species differences: RF/6A cells are derived from rhesus monkeys, and HUVECs are derived from humans. The mild decrease in the amount of VEGF secreted by HUVECs at 48 h was possibly due to the significant decrease of cell numbers. Moreover, it is reported that VEGF binding to fibronectin has a function in angiogenesis [43]. As noted above the downregulation of fibronectin could lead to the degradation of the capacity of VEGF to act as a promoter of angiogenesis. However, the short time span used in collecting the cells and supernatants limited the experimental data to that which reflected early changes in cell factor levels. With this in mind, we cannot exclude the possibility that the VEGF level changes in later stages through a negative feedback induced by hypofunction of vascular endothelial cells and related signaling pathways. Thus, we can only conclude that VEGF is not influenced by upregulation of HTRA1 during the initial lesion formation stages. Platelet derived growth factor (PDGF) are also vital elements of neoangiogenesis. Inhibitors targeting both VEGFR and PDGFR can contribute to the regression of neovascular vessels in tumors [44]. However, neovascularization in DR and AMD may differ significantly from angiogenesis in other pathological processes such as tumor angiogenesis [45]. Jo et al. have demonstrated that a combination therapy with anti-VEGF and anti-PDGFR is more effective for CNV prevention and regression [46].Nevertheless, in a model of injury-induced choroidal neovascularization, a VEGF inhibitor decreases CNV but administration of a PDGF inhibitor does not decrease CNV [47]. Moreover, PDGFR do not act on vascular endothelial cells, but on pericytes. PDGF induces pericyte recruitment and supports vascular maturation. HTRA1 have no effect upon the expression and activity of PDGFR in RF/6A cells and the role of HTRA1 on neovascularization may need further study on other cells, such as pericytes.

In summary, the results of our study indicate that HTRA1 rs11200638 variants are a risk factor for PCV. Upregulation of HTRA1 expression in RF/6A cell and HUVECs inhibited their proliferation, migration, tube formation, and fibronectin expression, whereas laminin, VEGF and MMP-2 expressions were not demonstrably affected. This study highlights the important role that HTRA1 may play in the initial formation of vascular lesions, and provides a better understanding of the effect of HTRA1 on PCV pathogenesis.

## Supporting Information

Table S1 **General characteristics of case-control studies included in meta-analysis.**
(DOC)

Table S2 **Analysis of the HTRA1 gene polymorphisms and PCV.**
(DOC)

Figure S1 **Meta-analysis of HTRA1 rs11200638 polymorphism and PCV.** Forest plot for meta-analysis of association between HTRA1 rs11200638 G>A polymorphism and PCV risk. Each study is shown by the point estimate of the odds ratio (OR) and 95% confidence interval (CI) for the OR.
(TIF)
