# Supplementary material for: Overexpression of HTRA1 Leads to Down-Regulation of Fibronectin and Functional Changes in RF/6A Cells and HUVECs
Source: PLoS One. 2012 Oct 8;7(10):e46115. doi: 10.1371/journal.pone.0046115 (PMC3466263; doi:10.1371/journal.pone.0046115)
Supplement: Table S1 — General characteristics of case-control studies included in meta-analysis. (DOC) [file pone.0046115.s001.doc]

Table S1 General characteristics of case-control studies included in meta-analysis

|  |  | Genotype distribution | | | | | | |  | | |
| --- | --- | --- | --- | --- | --- | --- | --- | --- | --- | --- | --- |
|  |  | Case, n=432 | | |  | Control, n=575 | | |  | HWE (p value) | |
| First author, year | country | AA | AG | GG |  | AA | AG | GG |  | Case | Control |
| Kondo 2007 | Japan | 32 | 32 | 12 |  | 16 | 40 | 38 |  | 0.71 | 0.63 |
| Lee 2008 | Singapore | 32 | 28 | 12 |  | 15 | 52 | 26 |  | 0.41 | 0.43 |
| Gotoh 2011 | Japan | 67 | 75 | 39 |  | 33 | 143 | 100 |  | 0.13 | 0.24 |
| Park 2011 | Korean | 51 | 40 | 12 |  | 15 | 48 | 49 |  | 0.64 | 0.84 |

HWE= Hardy-Weinberg equilibrium.
