# Supplementary material for: Overexpression of HTRA1 Leads to Down-Regulation of Fibronectin and Functional Changes in RF/6A Cells and HUVECs
Source: PLoS One. 2012 Oct 8;7(10):e46115. doi: 10.1371/journal.pone.0046115 (PMC3466263; doi:10.1371/journal.pone.0046115)
Supplement: Table S2 — Analysis of the HTRA1 gene polymorphisms and PCV. (DOC) [file pone.0046115.s002.doc]

Table 2 Analysis of the HTRA1 gene polymorphisms and PCV.

|  | AA vs GG | |  | AG vs GG | |  | A vs G | |
| --- | --- | --- | --- | --- | --- | --- | --- | --- |
| Studies | OR | 95%CI |  | OR | 95%CI |  | OR | 95%CI |
| Kondo | 6.33 | 2.62~15.33 |  | 2.53 | 1.14~5.63 |  | 2.76 | 1.78~4.30 |
| Lee | 4.62 | 1.84~11.58 |  | 1.17 | 0.51~2.66 |  | 2.24 | 1.44~3.51 |
| Gotoh | 5.21 | 2.98~9.09 |  | 1.34 | 0.85~2.14 |  | 2.24 | 1.71~2.94 |
| Park | 13.88 | 5.91~32.62 |  | 3.40 | 1.59~7.26 |  | 4.15 | 2.77~6.22 |
| Total | 6.43 | 4.42~9.33 |  | 1.75 | 1.27~2.42 |  | 2.64 | 2.20~3.17 |

PCV=polypoidal choroidal vasculopathy; CI=confidence interval; OR= odds ratio.
