# Supplementary figures and images for: Overexpression of HTRA1 Leads to Down-Regulation of Fibronectin and Functional Changes in RF/6A Cells and HUVECs
Source: PLoS One. 2012 Oct 8;7(10):e46115. doi: 10.1371/journal.pone.0046115 (PMC3466263; doi:10.1371/journal.pone.0046115)

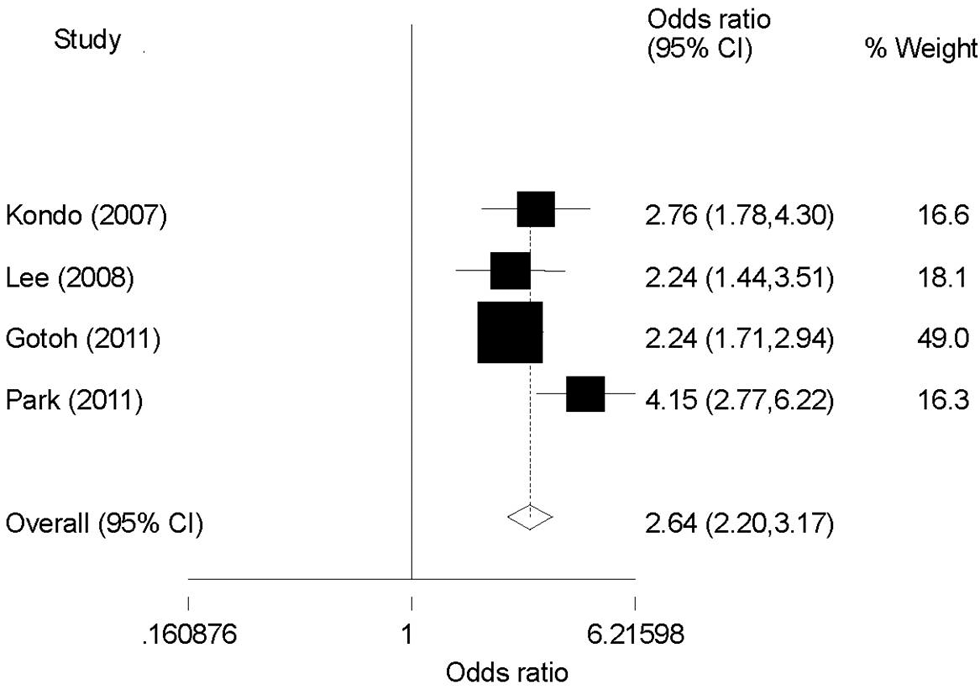

Supplement: Figure S1 — Meta-analysis of HTRA1 rs11200638 polymorphism and PCV. Forest plot for meta-analysis of association between HTRA1 rs11200638 G>A polymorphism and PCV risk. Each study is shown by the point estimate of the odds ratio (OR) and 95% confidence interval (CI) for the OR. (TIF) [file pone.0046115.s003.tif]
